# A boost in self-esteem after positive social evaluation predicts social and non-social learning

**DOI:** 10.1098/rsos.230027

**Published:** 2023-05-24

**Authors:** Charlotte van Schie, Jennifer L. Cook, Bernet Elzinga, Verena Ly

**Affiliations:** ^1^ Department of Clinical Psychology, Leiden Institute for Brain and Cognition, Leiden University, Leiden, The Netherlands; ^2^ Institute Office, Institute of Psychology, Leiden University, Leiden, The Netherlands; ^3^ School of Psychology, University of Birmingham, Birmingham, UK; ^4^ Illawarra Health and Medical Research Institute and the School of Psychology, University of Wollongong, Wollongong, Australia

**Keywords:** self-esteem, social learning, social acceptance, social rejection, self-verification

## Abstract

Fluctuations in self-esteem resulting from social acceptance and rejection could guide social behaviour by putting us in a state that is more or less open to social experiences. However, it remains unclear whether social acceptance and rejection may shape learning from social information depending on individual differences in self-esteem changes. Here we used a social feedback paradigm to manipulate social acceptance and rejection in a between-subjects design. Subsequently, we administered a behavioural task that enables the assessment of how well individuals learn on the basis of own experiences versus social information. Participants receiving positive (*N* = 43) versus negative (*N* = 44) social evaluation demonstrated an increase in subjective self-esteem. Importantly, the effect of the social evaluation on social learning was moderated by self-esteem changes. Specifically, an increase in self-esteem, as induced by positive evaluation, was associated with increased learning from social, but decreased learning from individual information. A decrease in self-esteem in response to negative evaluation was associated with decreased learning from individual information. These data suggest that increases in self-esteem in response to positive evaluation can induce a shift in the inclination to use social versus non-social information and may open one up to constructive learning from others.

## 1. Introduction

In a wide range of decisions, including everyday choices as what to wear and larger decisions around which job to apply for, we take social advice into account. Such instrumental decisions are guided by information coming from personal experiences of reward and punishment, as well as information from our social partners [1,2]. It has been theorized that social learning is evolutionarily adaptive, as it allows us to accumulate large amounts of complex knowledge in an efficient way across generations [2–4]. Indeed, observational learning, complex imitation, understanding of social cues and theory of mind play an important role in the human acquisition of cognitive skills and knowledge [5]. From a decision theoretic perspective, the ability to consider social information in the decision-making process is beneficial as this allows more optimal choice selection [6,7].

To make decisions, people combine signals derived from two parallel processes that support learning from social and personal reward-based information [6,8]. For optimal decision-making, it is important to be able to flexibly learn from social information and personal experiences allowing us to adaptively interact with the environment [9,10]. For instance, it may be relevant to adapt the tendency toward incorporating social information in decisions depending on the qualities of the source providing the social information (e.g. trustworthiness, intentions) [6,11]. Previous interaction experiences can foster the propensity to flexibly shift between relying on one's own perception and using social information. Importantly, the type of social interactions people have had, now or in the past (e.g. positive, negative), may put us in a state that is more or less open to learning from others [12]. However, it remains largely unknown how common consequences of daily social interactions, such as positive versus negative social experiences, influence the way in which individuals learn from social information. Research on this topic is relatively scarce, and—as we will outline below—the literature concerning the effects of positive and negative social experiences on social processing is rather inconsistent. To fill this gap, the main aim of the current study is to investigate how social acceptance versus rejection, manipulated with a social feedback paradigm, influences social learning.

A desire to belong and connect to others is thought to drive people to continuously monitor social interactions for cues that are indicative of acceptance and rejection [13–15]. People can pick up cues of acceptance or rejection through the feedback they receive from others based on their personal characteristics such as being thought of as charming or arrogant. Such social monitoring could play an important role in guiding our behaviour in a social environment [16–18]. It has been shown that social rejection motivates behaviours that aim to increase acceptance and strengthen interpersonal connection [15]. After social rejection, individuals may show increased social attentiveness and sensitivity to social cues [13,19], prosocial behaviour [20,21] and a preference for social interactions [22–24]. Additionally, one study showed that individuals reduced reliance on *misleading* social information in their decision-making after social rejection, but not after social acceptance, resulting in better performance in a social context [25]. Hence, social rejection can increase the sensitivity to social information which may be beneficial in a decision-making context.

While findings support increased social information processing following social rejection, other findings support reduced openness to social information. Social rejection can lead to interpersonal distrust and hence withdrawal, especially when the rejection is more chronic and severe [15,26–30]. For instance, rejected individuals may be inclined to avoid perpetrators of social rejection and even social interactions in general. It has also been shown that rejected individuals may react with aggression towards those who rejected them [31].

In addition to these contradicting findings on the effect of social rejection, there is a dearth of studies on how social acceptance influences social connections and social information processing. There is some evidence that social acceptance may foster trust in others [32] and enhance social information processing [33]. A functional magnetic resonance imaging study found that when people's positive self-views are confirmed, they may be more open to others indicated by a greater liking of the person that provided feedback indicative of social acceptance [34].

One aspect that could aid in reconciling the seemingly opposing findings and understanding these varied behavioural responses to social evaluation, is that these behaviours may depend on individual differences in reactions to social evaluations that can be measured as changes in state self-esteem [15,34–37]. Fluctuations in self-esteem in response to social evaluation are thought to guide our behaviour in a social context [16–18]. For example, some people may internalize the rejection and show a decrease in self-esteem after social rejection. This reaction has been referred to as a breaking response where the individual lowers their self-expectations to be in line with rejection [38]. People who experience a decrease in self-esteem after social rejection will feel the need to reconnect and be open to others (e.g. [17]). On the other hand, people may reconstrue the rejection so that it is less of a threat to the self, i.e. when the rejection is attributed externally and self-esteem increases. An increase in self-esteem after rejection could be part of a compensating reaction that results in evaluating others more negatively [38]. Hence, an increase in self-esteem after rejection may reduce the openness to social information. Regarding social acceptance, previous studies have shown that when positive evaluations had an uplifting effect, such as when it confirms people's self-views, others are evaluated more positively [32,34]. Therefore, social acceptance, particularly when it increases self-esteem, may be related to being more open to social information.

Thus, the effects of positive and negative social evaluations on one's openness to social information could depend on individual differences in how self-esteem changes in response to social evaluation. Specifically, the effects of rejection (versus acceptance) on social information use could be moderated by self-esteem. To investigate this idea directly, this study set out to assess the effects of social evaluation (positive versus negative) on social learning (versus individual learning). We were interested in the role of changes in state self-esteem in response to rejection and acceptance to understand if this may relate to the degree of social learning [17,38]. We used a modified version of a social feedback paradigm to manipulate social acceptance and rejection between subjects [34,35]. During this manipulation, participants received either standardized positive or negative social feedback, which was supposedly based on their personal characteristics. Subsequently, participants performed a social-learning task which enabled the assessment of learning from social versus individual information [6,39].

Consistent with previous findings using social feedback paradigms [34,35], we expected that overall the group receiving positive social evaluations would show an increase, whereas the group receiving negative social evaluations would show a decrease in self-esteem. Critically, we expected an interaction effect where the individual differences in the degree of self-esteem change after social rejection and acceptance relate to individual differences in social learning [38]. In line with literature pointing to increased efficiency in social information processing after social rejection [13,19], it could be hypothesized that negative (versus positive) social evaluations would increase the reliance on social information, specifically in those who react with a *decrease* in self-esteem. Conversely, negative (versus positive) social evaluations may decrease social learning [15,33], in those who react with an *increase* in self-esteem. Finally, positive (versus negative) social evaluations may increase social learning, specifically in those who react with an increase in self-esteem [32]. This study was restricted to women based on findings demonstrating increased response to social evaluation in women [40,41].

## 2. Material and methods

All materials, data and codes can be found on OSF: https://doi.org/10.17605/OSF.IO/PZT47.

### 2.1. Participants

We originally planned to include ninety participants based on our previous effects using versions of the social feedback paradigm (see below), but due to availability of resources we managed to recruit eighty-eight students and recent graduates from Leiden University. Due to technical problems, one participant (from the positive feedback condition) could not perform the social-learning task and was therefore excluded from the analyses. Thus, the final sample consisted of 87 female participants (*M*_age_ = 21.91, s.d. = 3.36, range = 18–30 years). All participants demonstrated normal or corrected-to-normal visual acuity and sufficient level of English in speaking and reading. Exclusion criteria were current neurological and psychiatric disorders, regular use of medication (except for contraceptives), use of psychotropic drugs, pregnancy or being under the influence of alcohol or the aftermath thereof. All participants gave written informed consent and received payment or course credits as a reimbursement for participation. The study was approved by the internal Psychology Research Ethics Committee of Leiden University (CEP17-0516/208).

### 2.2. General procedure

Participants were screened for the exclusion criteria via a questionnaire that was sent by e-mail. After screening and inclusion, one experimental session took place at the laboratory of Leiden University. In a between-subjects design, participants were randomized to receive either positive or negative social feedback from a ‘social feedback provider’ (confederate 1) during the social feedback paradigm. Subsequently, participants performed the social-learning task, which assessed how much individuals learned from social information, and individual information (individual experience based on outcome history). In this learning task, participants received social advice supposedly given by a ‘social advisor’ (confederate 2). To increase the credibility of the procedure, participants were introduced to two people at the start of the session who in reality were confederates to the study. They were informed that they would be partaking together in the study with different roles that were supposedly decided on the spot. See electronic supplementary material, S1, for more details about the general procedure. State self-esteem was assessed before and after the social feedback paradigm, as well as after the social-learning task to determine immediate and delayed changes in self-esteem in response to the feedback compared to baseline. The session concluded with an exit interview to check whether the participant found the experimental set-up credible. Finally, participants were fully debriefed about the study.

### 2.3. Material

#### 2.3.1. Social feedback paradigm

**Figure 1.**
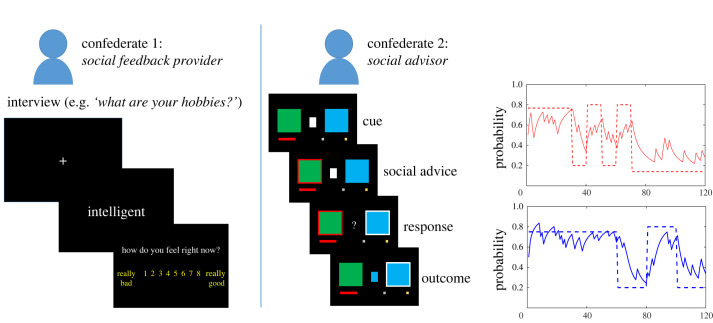
Social feedback paradigm (left). Participants received social feedback on a screen putatively coming from a social feedback provider (confederate 1) based on a personal interview. Social-learning task (right). After the personal social feedback, participants performed the social-learning task, which indexes learning from social information putatively given by a social advisor (confederate 2) versus learning from individually experienced non-social information (outcome history). *Example:* social (*red*) and individual (*blue*) Bayesian learner models. Red dashed lines represent programmed probabilities of correct social information and blue dashed lines of the blue choice being correct; solid lines represent estimated probabilities underlying the programmed sequences.

The social feedback paradigm was a modified version of existing social feedback paradigms [34,35]) (figure 1 left) and was validated in an independent sample (see electronic supplementary material, S2). The current version involved a between-subject design where participants received either standardized positive or standardized negative evaluative feedback. Specifically, participants were informed that in the context of an investigation about forming impressions, they would receive feedback that was given by another participant, the social feedback provider, who in reality was the first confederate. Participants were informed that the social feedback provider would form an impression about them based on a personal interview and would indicate their feedback by choosing from a list of adjectives. After this instruction and prior to receiving the pseudo feedback, the participants were interviewed individually without the confederates in the room (for approx. 5 min). The interview was composed of personal questions and three self-relevant moral dilemmas that would invite participants to discuss both positive and negative characteristics of themselves so that both positive and negative social feedback could be suitable. The interview was recorded with a voice recorder which was then supposedly given to the social feedback provider to form their evaluative feedback. Participants then waited approximately 8 min before the feedback was presented, ostensibly for the social feedback provider to listen to the interview and provide feedback.

Participants were shown the standardized feedback one word at the time on the computer screen in front of them. In each trial, a fixation cross appeared in the middle of the screen for 500 ms followed by the feedback word stimulus for 2500 ms. To assess mood changes upon feedback participants were required to rate ‘How do you feel right now?’ on an 8-point Likert scale (ranging from 1 = *really bad* to 8 = *really good*), after offset of the word stimulus. See figure 1 (left) for an illustration of the trial procedure. The task was run using E-prime 2.0.

The negative social feedback condition received 9 negative and 9 intermediate words; whereas the positive social feedback condition received 9 positive and 9 intermediate words. The same intermediate words were used in both social feedback conditions. Intermediate words were included in the feedback to balance out the intensity of the social feedback and increase overall credibility. Examples of feedback words included: ‘serious’ (intermediate), ‘interesting’ (positive) and ‘lazy’ (negative). The order of the words was randomized in each social feedback condition and words with the same valence did not appear twice in a row. See electronic supplementary material, S1, for more details about the validation of the word stimuli in an independent sample.

#### 2.3.2. Social-learning task

To enable assessment of the effects of the social feedback paradigm and state self-esteem changes on social learning, we administered a social-learning task after the social feedback paradigm. This social-learning task has been validated in previous studies and enables the assessment of how well individuals learn on the basis of own experiences versus social information (figure 1 right) [6,39,42]. The goal of this task is to earn as many points as possible by optimally using information based on own experiences (outcome history) and the information supposedly coming from the social advisor, which in reality was based on pseudorandomized schedules. Specifically, the task requires participants to indicate at each trial whether the blue or green stimulus is the likely correct choice by combining information from two sources: (1) the participant's own experience of rewards after choosing the blue or green stimulus and (2) the social advice and the likelihood of this being correct based on the history of advice utility. At each trial, the participant could use the outcome to learn—in parallel—about the likelihood of a stimulus being the correct choice, and the correctness of the social advice. Throughout the task, the probability that the blue stimulus is rewarded and the probability that the advice is rewarded were manipulated independently. Thus, optimal performance in this task required participants to track these two probabilities and combine them into an overall probability of the correct response.

*Instruction.* Participants were instructed that either the blue or the green stimulus would be correct on each trial and that the correct choice would be rewarded. Participants were further instructed that the probability of the blue (or green) stimulus being rewarded and of the advice being rewarded would change over time. However, they were not informed about the nature of how the probabilities would change over time. Moreover, participants were informed that the social advisor had been practicing multiple rounds of this social-learning task. Thus, the social advisor in the social-learning task was depicted as someone who could provide an expert opinion based on prior training. However, we warned the participant that the computer had mixed up the order of the advisor's trials, such that the utility of the advice varies over phases. Participants were informed about their performance through a red bar presented on the screen across the whole task, of which the length was proportional to the acquired points. Depending on the participant's performance, the red bar could reach the ‘silver’ or ‘golden’ target at the end of the task indicating the fictional prize.

*Trial procedure.* In each trial, a blue and a green cue appeared on the screen for 1–4 s. After this cue presentation phase, one of the two stimuli was marked by a red frame (0.5–2 s) indicating to participants that this is the correct response according to the social advisor. After this advice phase, participants were required to make a choice between the two cues. The participant's response was followed by a delay (0.5–2 s), after which the correct choice was displayed by a green or blue block in the middle of the screen (1–3 s) as feedback (figure 1).

*Pseudorandomized objective probability schedules.* The probability of the blue or green stimulus being correct (reward history) and the likelihood of social information being correct varied according to four pseudorandomized schedules (A, B, C, D) exactly as used in previous work [39]. Participants received one of the four schedules consisting of 120 trials in total. For the randomization schedule A, the reward history was stable during the first 60 trials, with a 75% probability of blue being correct. During the next 60 trials, the reward history was volatile, switching every 20 trials between 80% green being correct and 80% blue being correct. Meanwhile, during the first 30 trials, the social information was stable, with 75% of choices being correct. During the next 40 trials, the social information was volatile, switching every 10 trials between 80% incorrect and 80% correct. During the final 50 trials, the social information was stable again, with 85% of choices being incorrect. Schedules B, C, D were inverted and counterbalanced versions of schedule A (see electronic supplementary material, S3 figure S1). The schedules were counterbalanced between the positive versus negative feedback condition, to exclude the possibility that any effects of the social feedback could be explained by differences in the pseudorandomized schedules.

The social-learning task was controlled by the PC running Psychophysics Toolbox Version 3 with Matlab version R2016a.

#### 2.3.3. Subjective reports and manipulation check

We used several subjective reports in this experimental procedure. First, we assessed state self-esteem at three time points to measure changes throughout the procedure. Second, age, social support, socioeconomic status, social dominance, trait self-esteem and fear of negative evaluation were assessed at the beginning of the experiment, because these factors have been related to individual differences in social connectedness and response to social feedback or social learning [34,39,43–45]. We included these measures to test for any prior differences between the social feedback conditions on these potential confounding variables. Finally, as manipulation check, participants were asked about their experience of the social feedback paradigm and the confederates^1^.

##### 2.3.3.1. State self-esteem

State self-esteem was measured using the item: ‘How satisfied do you feel with yourself right now?’ and was rated using a Visual Analogue Scale ranging from *0 = not at all satisfied* to *100 = very satisfied.* This state self-esteem item was derived from Eisenberger *et al*. [35] and was validated in a separate study (see electronic supplementary material, S2). State self-esteem was assessed before and after the social feedback paradigm, as well as after the social-learning task to determine immediate and delayed changes in self-esteem in response to the feedback compared to baseline.

##### 2.3.3.2. Trait self-esteem

The Rosenberg self-esteem scale is a widely used measure to assess trait self-esteem [46]. Participants responded to ten items on a four-point Likert scale to indicate the extent to which they feel positive or negative about themselves in general. This questionnaire demonstrated good reliability and validity [47,48].

##### 2.3.3.3. Social support

The Multidimensional Scale of Perceived Social Support was used to assess the participants' level of perceived social support from three different sources: family, friends and significant other [49]. Participants responded to 12 items on a seven-point Likert scale.

##### 2.3.3.4. Socioeconomic status

Socioeconomic status was determined using the Barratt Simplified Measure of Social Status [50]. This measure assesses socioeconomic status using educational and occupational information about participant, spouse and parents. The scoring was adjusted to the Dutch educational system. Educational status was measured on a seven-point measure.

##### 2.3.3.5. Dominance questionnaires

We measured social dominance using the social dominance scale containing nine statements [43]. Participants are asked to indicate how much the statement applies to them by scoring themselves on a six-point Likert scale.

##### 2.3.3.6. Fear of negative evaluation

Fear of negative evaluation is defined as: ‘An apprehension about others’ evaluations, distress over negative evaluations by others, and the expectations that others would evaluate one negatively’ [51]. A revised version of the Brief Fear of Negative Evaluation scale (BFNE-R) was used to assess social anxiety in the current study [52]. It consists of 12 items and requires participants to indicate on a 5-point Likert scale how characteristic each statement is of them. Strong empirical evidence exists to support both the validity and reliability of the BFNE-R [52].

##### 2.3.3.7. Manipulation check

To assess if there was any existing doubt about the experiment among participants, the experimenter conducted an exit interview at the end. General questions were asked about the believability of the confederate and the feedback that the participants received. The inclusion of this allowed for two separate analyses to be conducted: a believers-only of the experimental manipulation analysis, in addition to the full sample analysis.

### 2.4. Data and statistical analyses

#### 2.4.1. Demographic data and the social feedback paradigm

We used analysis of variance (ANOVA) to test for any differences between the positive and negative feedback groups in terms of social support, socioeconomic status, social dominance, trait self-esteem and fear of negative evaluation.

#### 2.4.2. Social feedback and its influence on mood and state self-esteem

We first tested whether participants experienced lower mood during the social feedback paradigm in the negative versus the positive social feedback condition and particularly lower for negative words compared to intermediate words and better for positive words compared to intermediate words. To this end, we ran a multi-level model with mood ratings for each of the feedback words as outcome and as predictors the feedback condition (negative/positive) on the second level, valence of the word (intermediate/emotional (positive and negative)) on the first level and the valence by condition interaction. The intermediate valence of words was set as the reference category. χ^2^ tests were used for model comparisons to understand whether added effects were a better fit to the data than a previous model without the added effects. Second, the effects of the social feedback paradigm on state self-esteem were tested. We performed a multi-level analysis with state self-esteem ratings as outcome and as predictors the feedback condition (negative/positive) on the second level, time of rating (baseline/immediate/delayed) on the first level and the condition by time interaction. The time at baseline was set as the reference category.

#### 2.4.3. The influence of the social feedback paradigm on social and individual learning

Optimal performance in the social-learning task would require learning a probability associated with blue being correct based on the outcome history, and a probability of the social advisor giving correct advice based on the history of correct advice. These probabilities should then be combined to guide the decision. Following procedures from previous work [6,39], a Bayesian learner model algorithm was used to create a model for optimal individual learning and a model for optimal social learning. Critically, these two models have successfully predicted performance on the social-learning task demonstrating that the models explain a significant amount of variance in participants' performance on the task [6]. The algorithm used to create these models has been documented in detail by Behrens *et al.* [6].

The model assumes that outcomes (e.g. blue being rewarded) are generated with an underlying probability, *r* (e.g. the probability that blue is rewarded). The goal of the model is to predict *r* on the upcoming trial (i.e. predict *r_i_*_+1_). This is achieved in a Markovian fashion where, when an outcome is observed, the new outcome probability (e.g. probability that blue is rewarded on the next trial) depends only on this observed outcome and on the previous outcome probability, not on the full history of previous outcome probabilities. The changeability of *r* is represented by *p*(*r_i_*_+1_|*r_i_*), i.e. the probability that *r* will change from the value *r_i_* to the value *r_i_*_+1_ over the course of a single trial. We represent this as a beta probability distribution: *p*(*r_i_*_+1_|*r_i_*, *v*)∼*β* (*r_i_*, *V*), where *r_i_* and *V* = exp(*v*)^2^ define the mean and width of the distribution respectively. A large value of *v*—which we refer to as ‘volatility’—leads to a wide distribution, implying *r* may be expected to change greatly (as in a volatile environment wherein the rewarded option flips between blue and green); a small value of *v* leads to a narrow distribution, implying *r* may be expected to change very little (as in a stable environment where, for example, the blue option is typically rewarded). Mathematically, we track the changing volatility, *v_i_*, in the same manner as the reward rate, *r_i_*, by again assuming a Markovian progression. The changeability of *v* is represented as *p*(*v_i_*_+1_|*v_i_*). Here, the distribution does not need to be constrained between 0 and 1, so the form of the transitional distribution can be taken to be Gaussian: *p*(*v_i_*_+1_|*v_i_*, *k*)∼*N* (*v_i_*, *K*), where *K* = exp(*k*) controls the rate of change of volatility. A large value for *k* leads to a wide transitional distribution, and would describe an environment that moves quickly between stable and volatile periods; a small value for *k* leads to a narrow distribution and would describe an environment whose volatility could only change slowly.

The generated individual learning model provides the estimated trial-by-trial reward probability of the blue choice. The social learning model provides the estimated trial-by-trial probability of correct social information. Using this social learning model, we first computed trial-by-trial values by combining the probabilities with the social advice on each trial such that the value reflected the probability of a blue choice if social learning was optimal (e.g. if the probability that the social information is correct is 0.8 and the advice is blue, the probability of selecting blue based on social information should be 0.8, but the probability of selecting green based on the advice should be 0.2).

Following previous procedures [6,39], we used logistic regression to determine the degree to which participants' choices were explained by the optimal individual learner model and the optimal social learner model. Specifically, we used multi-level analysis with the logit function. The two optimal learner models were set as predictors on the first level and regressed against the choice data (where blue is 1 and green is 0). Higher parameter estimates of the individual and social learner models indicate more Bayes optimal learning from individual and social information respectively. The immediate change in self-esteem (Δself-esteem) from prior to after the social feedback manipulation was included as a predictor on the second level. Standardized values of the optimal learner models and Δself-esteem were used in all analyses. Additionally, social feedback condition was included as a predictor on the second level, with the negative feedback condition as reference category. In sum, logistic multi-level analysis was used with choice per trial (0/1) as outcome and social feedback condition (positive/negative), learner model (social/individual) and Δself-esteem as predictors.

To test if social feedback condition influences social learning and whether or not these effects depend on changes in self-esteem, we were particularly interested in the two-way (condition × learner model) and three-way (condition × learner model × Δself-esteem) interactions respectively. Therefore, we constructed 7 models of increasing complexity where the most complex model (M7) included all main, two-way, and three-way interaction effects and was the model of interest (table 1). Model comparisons were conducted with the χ^2^ test to understand whether added effects were a better fit to the data than a previous model without the added effects. In all steps where main or interaction effects were added involving learner models, the terms with social and individual learner models were added simultaneously to test the specificity of social feedback on social learning. Finally, to control for individual differences in baseline state self-esteem as measured prior to the social feedback paradigm, the same analyses were conducted with baseline state self-esteem as a predictor on the second level of no interest.

**Table 1.** Model comparisons of the role of self-esteem change in social learning paradigm.

model	no. par	AIC	BIC	log likelihood	*χ*^2^ (df), *p*
M0: random intercept	2	14 399	14 413	14 395	
M1: + individual learner model + social learner model	4	12 989	13 018	12 981	$\chi_{2}^{2}=1413.45$, *p* < 0.001
M2: + feedback condition	5	12 988	13 024	12 978	$\chi_{1}^{2}=3.28$, *p* = 0.070
M3: + individual learner model × feedback condition + social learner model × feedback condition	7	12 984	13 035	12 970	$\chi_{2}^{2}=7.59$, *p* = 0.022
M4: + Δself-esteem	8	12 986	13 044	12 970	$\chi_{1}^{2}=0.74$, *p* = 0.391
M5: + Δself-esteem × feedback condition	9	12 987	13 052	12 969	$\chi_{1}^{2}=0.37$, *p* = 0.546
M6: + individual learner model × Δself-esteem + social learner model × Δself-esteem	11	12 966	13 045	12 944	$\chi_{2}^{2}=25.70$, *p* < 0.001
M7: + individual learner model × Δself-esteem × feedback condition + social learner model × Δself-esteem × feedback condition	13	12 920	13 014	12 894	$\chi_{2}^{2}=49.32$, *p* < 0.001

For all analyses, alpha was set at 0.05. Data from the social learning paradigm were preprocessed using MATLAB R2016a (The MathWorks, Natick, MA). Statistical analyses were performed using R v. 4.0.2 in R Studio v. 1.3.1093 with the packages lme4 for multi-level modelling [53,54]. For all effect parameters, bootstrapped 95% confidence intervals were calculated with 2500 simulations.

#### 2.4.4. Sensitivity analysis

Calculations for estimated power were performed using R with the fabs package (github\jbiesanz\fabs), which incorporates the uncertainties associated with effect size estimates. Initial sample size (*N* = 90) was based on an independent sample where a between-subject version of the social feedback task resulted in changes in self-esteem post-task (see electronic supplementary material, S2). This study found a two-way interaction effect of time × feedback condition on self-esteem $\left(\chi_{2}^{2}=41.81\right)$ with *N* = 444 observations from *N* = 111 participants. Based on this study, *N* = 261 observations from *N* = 87 participants would have an expected power of 0.98 to detect self-esteem change dependent on feedback condition. Our main interest is the 3-way interaction of feedback condition × learner model × self-esteem change in a multilevel model. We based estimates of expected power on the effects found in two studies. The study by Cook *et al*. [42] used the same social learning task and found a 3-way interaction (*F*_1,100_ = 8.171 with *N* = 102), albeit not within a multilevel model. Based on this study, *N* = 10 440 observations from *N* = 87 participants would have an expected power of 0.99 to find a similar effect. The study by Byrne *et al*. [25] used a similar design as in this study and found a 2-way interaction (*F*_1, 146_ = 10.95). Based on this study, *N* = 10 440 observations from 87 participants would have an expected power of 0.99 to find a similar effect.

## 3. Results

Based on the data from the manipulation check during the exit interview, almost all participants believed the roles that the confederates played (*N* = 86; 99%). Only one participant (1%) indicated that she did not believe the cover story of the study. Five participants (6%) indicated that they had some doubts about the validity of the social feedback retrospectively reported at the end of the experiment. Five out of these six participants were in the negative feedback condition. Analyses with and without these potential non-believers did not yield different results. Therefore, the analyses that we report are based on the whole sample including these potential non-believers.

### 3.1. Demographic data

Table 2 presents the demographic data. The reliabilities of the scales for social support, social dominance, trait self-esteem and fear of negative evaluation were high as indicated by the Cronbach's alpha (ranging from 0.79 to 0.95). The positive versus negative social feedback condition did not differ in age, social support, socioeconomic status, social dominance, trait self-esteem and fear of negative evaluation (all *F* < 1.33).

**Table 2.** Demographic data (mean and SEM) for the positive and negative social feedback conditions.

	social feedback				
	positive (*n* = 43)	negative (*n* = 44)	Cronbach's alpha	*F*	*p*-value
age (in years)	21.52 (0.50)	22.30 (0.52)		1.17	0.282
social support	5.90 (0.10)	6.02 (0.15)	0.89	0.42	0.521
socioeconomic status^a^	47.79 (2.20)	51.12 (1.90)		1.33	0.253
social dominance	3.79 (0.13)	3.83 (0.13)	0.79	0.06	0.816
trait self-esteem	30.50 (0.69)	31.05 (0.55)	0.83	0.38	0.538
fear of negative evaluation	32.93 (1.61)	34.81 (1.72)	0.95	0.64	0.260

^a^Data on socioeconomic status was missing for four cases.

### 3.2. Positive versus negative social feedback influenced mood state and increased immediate state self-esteem

**Figure 2.**
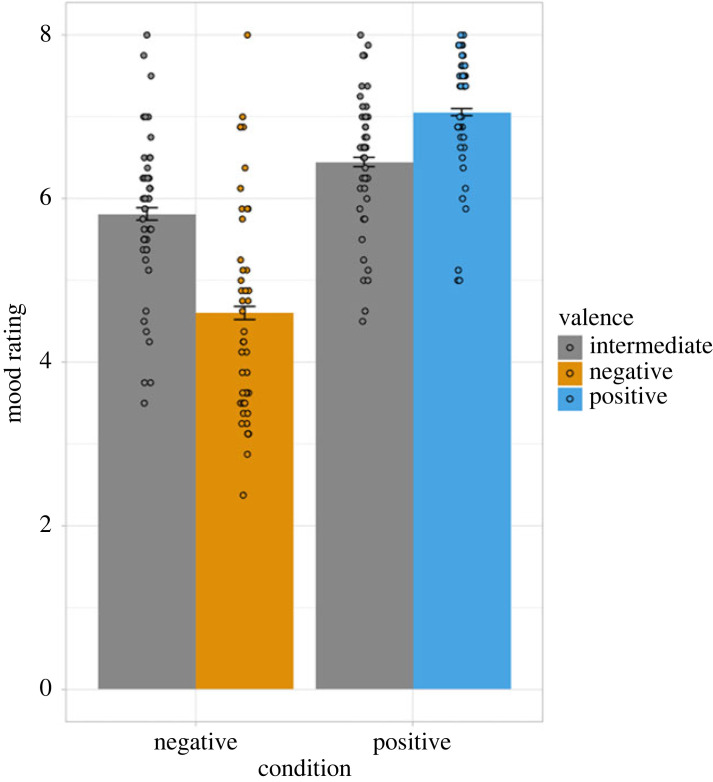
Average ratings for the word stimuli used in the social feedback paradigm for the negative(/intermediate) and positive(/intermediate) social feedback conditions. Participants rated ‘How do you feel right now?’ on an 8-point Likert scale (ranging from 1 = really bad to 8 = really good). The participants in the negative (versus positive) social feedback condition reported lower mood state during the social feedback paradigm. Error bars represent standard error of the mean. Dot points indicate the participants' mean mood rating by valence.

Multilevel analysis of the mood ratings during the social feedback paradigm revealed a main effect of valence ($\chi_{1}^{2}=25.58$, *p* < 0.001), a main effect of feedback condition ($\chi_{1}^{2}=46.92$, *p* < 0.001) and a significant valence × feedback condition interaction effect ($\chi_{1}^{2}=243.61$, *p* < 0.001; figure 2). In the negative feedback condition, negative compared to intermediate feedback words lowered mood (*b* = −1.21, s.e. = 0.08, *t* = −15.47, 95% CI [−1.36, −1.06]). In the positive feedback condition, positive compared to intermediate words heightened mood (*b* = 1.82, s.e. = 0.11, *t* = 16.37, 95% CI [1.60, 2.04]). Moreover, participants in the positive feedback condition had a better mood than participants in the negative feedback condition (*b* = 0.64, s.e. = 0.20, *t* = 3.12, 95% CI [0.23, 1.03]). For all cell means and standard deviations, see electronic supplementary material, S3 table S7.

**Figure 3.**
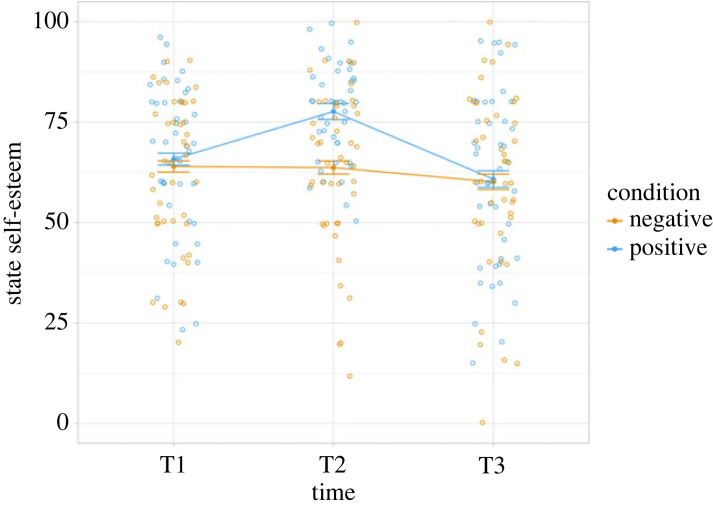
Changes in state self-esteem throughout the experiment. The social feedback task was provided between T1 (baseline) and T2 (immediate). The social-learning task took place between T2 (immediate) and T3 (delayed). Error bars represent standard error of the mean. Raw data points are plotted by time and condition.

Multilevel analysis of state self-esteem revealed a significant main effect of time ($\chi_{2}^{2}=28.21$, *p* < 0.001), no main effect of feedback condition ($\chi_{1}^{2}=2.33$, *p* = 0.127), and crucially, a significant feedback condition × time interaction effect ($\chi_{2}^{2}=16.90$, *p* < 0.001; figure 3). We observed that in the negative feedback condition, there was neither a change in state self-esteem immediately after the social feedback task (*b* = −0.27, s.e. = 2.45, *t* = −0.11, 95% CI [−5.10, 4.61]) nor a delayed change (*b* = −3.82, s.e. = 2.45, *t* = −1.56, 95% CI [−8.64, 1.05]) compared to baseline state self-esteem. In the positive feedback condition, however, we observed an increase in state self-esteem immediately after the social feedback (*b* = 12.11, s.e. = 3.49, *t* = 3.47, 95% CI [5.24, 19.00]). At the delayed interval state self-esteem had returned to baseline level in the positive feedback condition (*b* = −1.16, s.e. = 3.49, *t* = −0.33, 95% CI [−8.18, 5.60]). For all cell means and standard deviations, see electronic supplementary material, S3 table S8.

### 3.3. Effects of social feedback on social learning depends on self-esteem change

**Figure 4.**
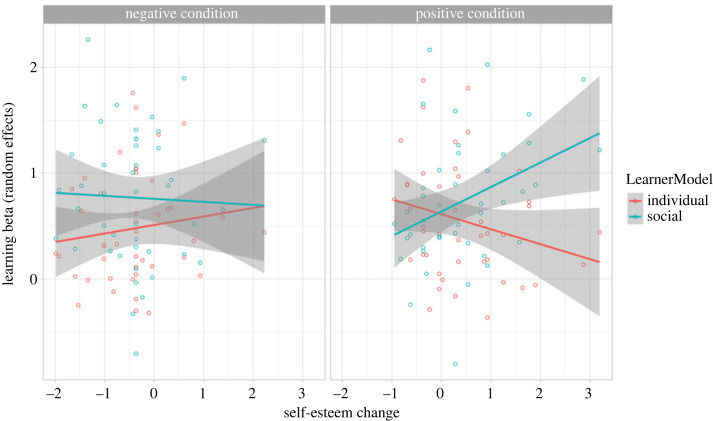
Individual and social learning as a function of self-esteem change after the negative (left panel) and positive (right panel) feedback condition. For illustration, learning betas were estimated as random effects of the social and individual learning model from model 1 reflecting the extent to which participant's choices were predicted by the optimal individual and social learning models respectively. Left panel: a decrease in self-esteem, as induced by the negative (versus positive) feedback, was associated with decreased individual learning. The degree of social learning was not dependent on self-esteem change in the negative condition. Right panel: an increase in self-esteem, as induced by the positive (versus negative) feedback, was associated with increased social learning and decreased individual learning.

Multi-level logistic analysis demonstrated that participants' choices were predicted by both optimal learner models (individual and social) (M1: $\chi_{2}^{2}=1413.45$, *p* < 0.001). In other words, choice behaviour was explained by both the Bayes optimal individual learner model and the Bayes optimal social learner model (individual learner model: *b* = 0.44, s.e. = 0.02, *Z =* 20.29, 95% CI [0.40, 0.48]; social learner model: *b* = 0.65, s.e. = 0.02, *Z =* 29.92, 95% CI [0.59, 0.68]). As expected, the social feedback condition by itself was not a significant predictor of choice behaviour, i.e. the main effect of social feedback condition (M2: $\chi_{1}^{2}=3.28$, *p* = 0.070). However, the degree to which choice behaviour was explained by the learner models depended on the feedback condition, i.e. feedback condition × learner models interaction (M3: $\chi_{2}^{2}=7.59$, *p* = 0.022). There was no main effect of Δself-esteem (M4: $\chi_{1}^{2}=0.74$, *p* = 0.391) nor was the two-way interaction of Δself-esteem × feedback condition predictive of choice behaviour (M5: $\chi_{1}^{2}=0.37$, *p* = 0.546). However, the change in self-esteem affected Bayes optimal learning from social and individual information, i.e. Δself-esteem × learner model interaction (M6: $\chi_{2}^{2}=25.70$, *p* < 0.001). Importantly, these effects also depended on individual differences in changes in self-esteem dependent on the feedback condition, as indicated by the significant three-way interactions: Δself-esteem × feedback condition × learner models (M7: $\chi_{2}^{2}=49.32$, *p* < 0.001). The complex three-way interaction model (M7) is the most likely model given the observed data (i.e. significant χ^2^ test indicates a better model fit compared to the previous model), even when considering the increased complexity (i.e. AIC does not increase compared to previous model). The three-way interaction is visualized in figure 4 and the effect parameters of this model (M7) are reported here. In this model, the negative feedback condition served as the reference condition. When participants in the negative feedback condition reported a decrease in self-esteem after the social feedback, they showed less Bayes optimal learning from individual information (individual learner model × Δself-esteem interaction: *b* = 0.07, s.e. = 0.04, *Z* = 1.98, 95% CI [0.003, 0.14]). Participants in the positive feedback condition of the social feedback task showed increased Bayes optimal learning from individual information in general (individual learner model × feedback condition interaction: *b* = 0.15, s.e. = 0.05, *Z =* 3.09, 95% CI [0.05, 0.25]). However, interestingly, when self-esteem increased following positive social feedback, learning from individual information was less Bayes optimal (individual learner model × feedback condition × Δself-esteem interaction: *b* = −0.21, s.e. = 0.05, *Z =* −4.30, 95% CI [−0.31, −0.12]; figure 4). Moreover, a higher increase in self-esteem after positive social feedback was associated with increased Bayes optimal learning from social information (social learner model × feedback condition × Δself-esteem interaction: *b* = 0.26, s.e. = 0.05, *Z =* 5.22, 95% CI [0.17, 0.37]).

Follow-up mixed effects logistic regressions conducted per feedback condition (for positive and negative feedback condition separately) suggest that the three-way interactions for social and individual learning were largely driven by the positive feedback condition. In the positive condition, the two-way interactions of Δself-esteem × individual learner model and Δself-esteem × social learner model were a significant addition to the model with main effects only ($\chi_{2}^{2}=70.26$, *p* < 0.001). It was found that an increase in self-esteem after positive social feedback was associated with less Bayes optimal learning from individual information (individual × Δself-esteem interaction: *b* = −0.14, s.e. = 0.03, *Z =* −4.20, 95% CI [−0.21, −0.07]), and more Bayes optimal learning from social information (social × Δself-esteem interaction: *b* = 0.23, s.e. = 0.03, *Z =* 6.68, 95% CI [0.17, 0.30]).

For exploratory purposes, we sought to understand whether the relative weight given to individual and social information was related to self-esteem change. To this end, we subtracted the individual learning beta from the social learning beta so that positive numbers reflect greater weight given to social information. In the negative condition, self-esteem change did not relate to relative weight given to social or individual learning (*r* = −0.14, 95% CI [−0.42, 0.17]). In the positive condition, more weight was given to social information as self-esteem increased (*r* = 0.38, 95% CI [0.09, 0.62]) (see electronic supplementary material, figure S2).

Finally, controlling for individual differences in baseline state self-esteem as measured prior to the social feedback manipulation yielded the same results in these main analyses (see electronic supplementary material, S3).

## 4. Discussion

In the current study, we tested the effects of social acceptance versus rejection on social learning. Specifically, we investigated whether individual differences in responding to the social evaluation as measured by changes in state self-esteem affected social learning from a new social partner. We manipulated social acceptance and rejection using a social feedback paradigm, in which participants received either standardized positive or negative feedback that was supposedly based on their personal characteristics. Subsequently, participants performed a social-learning task, where individuals could use both social information (advice provided by an expert) and individual information (individual experience based on outcome history) to optimize their performance on the task. Crucially, we demonstrated that the extent to which individuals use social versus nonsocial information in decision making is dependent on how they responded to rejection and acceptance, i.e. the degree to which self-esteem increased or decreased.

Specifically, we found that overall, positive (versus negative) social evaluation increased state self-esteem. A significant change in state self-esteem was not observed after negative social feedback. However, we observed a broad variety of changes in self-esteem as a response to the social evaluation. Interestingly, we found that the change in self-esteem after the positive (versus negative) social feedback predicted learning from social information. An increase in self-esteem, as induced by prior positive (but not negative) social feedback, was associated with *more* Bayes optimal learning from social information and *less* Bayes optimal use of individual information. Moreover, an increase in self-esteem following negative feedback was associated with increased use of individual information. These findings indicate that there are individual differences in the effect of social evaluation on social and individual learning and importantly, that these are dependent on the changes in self-esteem after negative and positive evaluation. We demonstrated these effects in a context where the social information was coming from a new social partner (rather than the accepting or rejecting interacting partner). These findings highlight the important influence that previous social experiences may have on new social experiences and are relevant to the decisions that we make in everyday life, which are more often than not preceded by social interactions.

Our data suggest that an increased self-esteem after positive social evaluations can increase subsequent social learning from a new social partner. This finding is contradictory to the literature indicating that social rejection rather than social acceptance increases the drive to socially (re)connect and the efficiency of social information processing [55]. Rather, this finding is reminiscent of previous literature suggesting that social acceptance increases trust in social partners who previously demonstrated social acceptance [32], though here we extend this to learning from a new social partner. Moreover, social acceptance has been shown to increase attention allocation to social cues [33]. In our current social-learning task, it could be that a greater response to social acceptance in terms of higher self-esteem increased attention allocation to the social advice, and promoted learning from this social information.

One way of understanding how receiving positive feedback may relate to increased attention allocation and openness to social information is through the process of self-verification. When people receive social feedback that others see them in the same way as they see themselves, it not only strengthens self-views but also fosters trust in others [56]. When the self-view is confirmed by others, mood and state self-esteem may heighten [34,57,58] and people may attend more to others [34,57]. This interpretation is in line with work demonstrating that attention allocation depends on the prospect of reward such that more attention is allocated to information with greater value [59,60]. Furthermore, it has been shown previously that prior beliefs about others’ abilities could influence social learning by affecting the level of attention toward the social information [61]. Thus, in the current study, those who showed a higher increase in self-esteem in the positive condition may have perceived their self-view to be more in line with the feedback, which could have increased the subjective value of information coming from others, even in a new context. Consequently, the social advice in the social-learning task could be weighed more heavily in decisions due to this increased subjective value of the social information in those who reported higher increase in self-esteem.

It may be that the response to the social evaluation in terms of self-esteem change also modulated the integration of social and individual learning of information. Our findings indicate that an increase in self-esteem following positive feedback was not only associated with more Bayes optimal use of social information but also with less Bayes optimal use of individual information. The opposing effect on social versus individual learning may be explained by an adjustment in the relative importance given to these two information sources. This could imply that preference is given to social information over individual information when the previous (positive) social feedback is seen as valuable (i.e. increase in self-esteem). Such interpretation is in line with previous work showing that social information is preferred when both personal and social information is reliable [62].

In the negative condition, we did not find opposing effects as the use of individual information increased, whereas the use of social information remained unchanged, when self-esteem increased after negative feedback. Our observations in the negative condition converge with the suggestion that—rather than reducing the tendency to use social information—individuals employ a strategy to update the weight of personal information and therewith indirectly adjust the relative importance of the two information sources [63]. This finding is consistent with previous literature showing that when social information is not reliable, a stronger preference is given to personal information without loss of weight of social information [63]. In the current study, people who received negative feedback and were able to increase their self-esteem may have been able to dismiss the social feedback as less reliable (i.e. a compensating response) [38]. This may have altered the perception of the utility of the social advice in a new context and increased the value of using individual information, at least temporarily.

However, further research is needed to replicate these findings and support these ideas. Future research could benefit from employing neuroimaging and computational modelling to investigate how responses to social feedback change the neural representations of social versus individual learning. There is a growing body of literature investigating how social information is acquired and integrated with individual information using computational modelling [6,11,61,64–66]. Such approaches could inform more directly how responses to social feedback influence social learning and the integration of social and individual information. For instance, these methods could enable one to tease apart and test if responses to social feedback have an impact on how social and individual information are combined through changing the weights of social versus individual information directly, through influencing higher-order arbitration between different (social) learning strategies, and/or through affecting higher-order processes related to the social partner [11,64,65].

An alternative, or perhaps additional, interpretation of the current findings is that both individual differences in responsiveness to positive social feedback in terms of changes in state self-esteem and the propensity to use social learning can be explained by individual differences in sensitivity/focus on social information. In other words, it could be that individuals, who are more sensitive to social information in the first place, are more likely to take social feedback to heart, and also learn better from social information. However, this explanation would have been more plausible if individuals who showed greater decreases in self-esteem from negative social evaluation also showed increased social learning in the social-learning task reflecting a higher sensitivity to social information. It is remarkable that the current effects are mainly observed in the positive social evaluation condition. Future studies are needed to be better able to draw conclusions about the direction of effects.

It may be somewhat surprising that we only found an overall change in state self-esteem for the positive feedback condition, but not for the negative feedback condition. Even though people were affected by the negative feedback in terms of lowered mood while receiving the feedback, this did not translate in lower state self-esteem after the task. However, as indicated by a meta-analysis [67], it is not uncommon that studies mainly find that social acceptance raises self-esteem, while social rejection has no effect. A reason for this is that the effects of social rejection on self-esteem might be muted by defensive processes [38,67] and there may be more individual differences in responding [15]. Here, we observed that it was not the negative evaluations in and of itself but the individual differences in response to the social evaluations, i.e. self-esteem change, that related to increased individual learning. A desire to connect or withdraw after social feedback may depend on how people responded to the social evaluations in terms of self-esteem change. This may also provide an explanation as to why some studies observed a motivation to connect whereas others observed withdrawal. Nevertheless, further research is needed to understand the effects of negative evaluations on social learning.

A limitation to the study is that we did not include a neutral-only control group. Since the effects of social acceptance are not necessarily the opposite or mirror image of social rejection [67], future research should include a neutral control group or include assessments of social learning at pre- and post-manipulation to allow more rigorous testing. The fact that five out of six participants who reported doubts about the credibility of the social feedback at the debriefing were in the negative feedback condition may indicate that the manipulation was less successful in this condition. However, another likely explanation is that these doubts about the negative feedback are an adaptive, compensating response, to the manipulation. That is, doubts about the procedure could be a mechanism to deal with the negative evaluation and a way to maintain the self-beliefs. It should be noted that overall most participants believed the feedback to be real and analyses with and without these participants did not change the results. Finally, we have investigated females only as males and females may value social feedback differently [40,41]. Future studies should include male participants and test for gender differences to understand how the findings translate to males.

## 5. Conclusion

Social experiences are vital to navigate daily life. Given the relevance of social learning, it is critical to better understand how positive versus negative social experience influences social learning. While previous work has mainly focused on how social acceptance/rejection influences a myriad of social processes, with inconsistent findings, here, we demonstrate a critical role of self-esteem change as a response to social evaluation in this relationship. This is the first study on social evaluation and subsequent social learning suggesting that increases in self-esteem in response to social acceptance are associated with more optimal social learning. The current findings underscore the important role of social experiences in the way we use social information in daily life decisions and may imply that positive interactions may open one up to constructive learning from others.

## Ethics

This study was conducted in accordance with the Declaration of Helsinki. All participants gave written informed consent and received payment or course credits as a reimbursement for participation. The study was approved by the internal Psychology Research Ethics Committee of Leiden University (CEP17-0516/208).

## Data Availability

All materials, data and codes can be found on OSF: https://doi.org/10.17605/OSF.IO/PZT47 [68]. The data are provided in electronic supplementary material [69].

## Footnotes

1 In addition to the described measures, we included the Liebowitz social anxiety scale, childhood trauma questionnaire, trait self-concept clarity and a self-esteem implicit association test. Participants also rated 90 adjectives on how well these adjectives described them. These measures are not relevant for the current study, and were therefore not analysed.

2 The exponential is taken so that integrals on the width parameter are performed in log space.

## References

[1] Boyd R, Richerson PJ. 1988 An evolutionary model of social learning: the effects of spatial and temporal variation. In Social learning: psychological and biological perspectives (eds TR Zentall, BG Galef), pp. 29-48. Hillsdale, NJ: Lawrence Erlbaum Associates, Inc.

[2] Boyd R, Richerson PJ, Henrich J. 2011 The cultural niche: why social learning is essential for human adaptation. Proc. Natl Acad. Sci. USA **108**, 10 918-10 925. (10.1073/pnas.1100290108) PMC3131818 21690340

[3] Schaik V, Burkart JM. 2011 Social learning and evolution: the cultural intelligence hypothesis. Phil. Trans. R. Soc. B **366**, 1008-1016. (10.1098/rstb.2010.0304) 21357223 PMC3049085

[4] Tomasello M. 2016 The ontogeny of cultural learning. Curr. Opin. Psychol. **8**, 1-4. (10.1016/j.copsyc.2015.09.008) 29506782

[5] Herrmann E, Call J, Hernández-lloreda MV, Hare B, Tomasello M. 2007 Humans have evolved specialized skills of social cognition: the cultural intelligence hypothesis. Science **317**, 1360-1367. (10.1126/science.114682) 17823346

[6] Behrens TEJ, Hunt LT, Woolrich MW, Rushworth MFS. 2008 Associative learning of social value. Nature **456**, 245-249. (10.1038/nature07538) 19005555 PMC2605577

[7] Burke CJ, Tobler PN, Baddeley M, Schultz W. 2010 Neural mechanisms of observational learning. Proc. Natl Acad. Sci. USA **107**, 14 431-14 436. (10.1073/pnas.1003111107) PMC2922583 20660717

[8] Rybicki AJ, Sowden SL, Schuster B, Cook JL. 2022 Dopaminergic challenge dissociates learning from primary versus secondary sources of information. Elife **11**, e74893. (10.7554/eLife.74893) 35289748 PMC9023054

[9] Corriveau KH et al. 2009 Young children's trust in their mother's claims: longitudinal links with attachment security in infancy. Child Dev. **80**, 750-761. (10.1111/j.1467-8624.2009.01295.x) 19489901

[10] Laland KN. 2004 Social learning strategies. Anim. Learn. Behav. **32**, 4-14. (10.3758/BF03196002) 15161136

[11] Diaconescu AO, Mathys C, Weber LA, Daunizeau J, Kasper L, Lomakina EI, Fehr E, Stephan KE. 2014 Inferring on the intentions of others by hierarchical Bayesian learning. PLoS Comput. Biol. **10**, e1003810. (10.1371/journal.pcbi.1003810) 25187943 PMC4154656

[12] Fonagy P, Allison E. 2014 The role of mentalizing and epistemic trust in the therapeutic relationship. Psychotherapy **51**, 372-380. (10.1037/a0036505) 24773092

[13] Gardner WL, Pickett CL, Brewer MB. 2000 Social exclusion and selective memory: how the need to belong influences memory for social events. Pers. Soc. Psychol. Bull. **26**, 486-496. (10.1177/0146167200266007)

[14] Pickett CL, Gardner WL, Knowles M. 2004 Getting a cue: the need to belong and enhanced sensitivity to social cues. Pers. Soc. Psychol. Bull. **30**, 1095-1107. (10.1177/0146167203262085) 15359014

[15] Williams KD. 2007 Ostracism. Annu. Rev. Psychol. **58**, 425-452. (10.1146/annurev.psych.58.110405.085641) 16968209

[16] Leary MR. 1999 Making sense of self-esteem. Curr. Direct. Psychol. Sci. **8**, 32-35. (10.1111/1467-8721.00008)

[17] Leary MR. 2005 Sociometer theory and the pursuit of relational value: getting to the root of self-esteem. Eur. Rev. Soc. Psychol. **16**, 75-111. (10.1080/10463280540000007)

[18] Leary MR, Tambor ES, Terdal SK, Downs DL. 1995 Self-esteem as an interpersonal monitor: the sociometer hypothesis. J. Pers. Soc. Psychol. **68**, 518-530. (10.1037/0022-3514.68.3.518)

[19] Bernstein MJ, Young SG, Brown CM, Sacco DF, Claypool HM. 2008 Adaptive responses to social exclusion: social rejection improves detection of real and fake smiles. Psychol. Sci. **19**, 981-983. (10.1111/j.1467-9280.2008.02187.x) 19000206

[20] Carter-sowell AR, Chen Z, Williams KD. 2008 Ostracism increases social susceptibility. Soc. Influ. **3**, 143-153. (10.1080/15534510802204868)

[21] Williams KD, Sommer KL. 1997 Social ostracism by coworkers: does rejection lead to loafing or compensation? Pers. Soc. Psychol. Bull. **23**, 693-706. (10.1177/0146167297237003)

[22] Lakin JL, Chartrand TL, Arkin RM. 2008 I am too just like you: nonconscious mimicry as an automatic behavioral response to social exclusion. Psychol. Sci. **19**, 816-822. (10.1111/j.1467-9280.2008.02162.x) 18816290

[23] Maner JK, Dewall CN, Baumeister RF, Schaller M. 2007 Does social exclusion motivate interpersonal reconnection? Resolving the ‘Porcupine Problem’. J. Pers. Soc. Psychol. **92**, 42-55. (10.1037/0022-3514.92.1.42) 17201541

[24] Williams KD, Cheung CKT, Choi W. 2000 Cyberostracism: effects of being ignored over the Internet. J. Pers. Soc. Psychol. **79**, 748-762. (10.1037/0022-3514.79.5.748) 11079239

[25] Byrne KA, Tibbett TP, Laserna LN, Carter-sowell AR, Worthy DA. 2016 Ostracism reduces reliance on poor advice from others during decision making. J. Behav. Decision Making **418**, 409-418. (10.1002/bdm.1886) PMC5410892 28469290

[26] Berg LJMVD, Tollenaar MS, Pittner K, Compier-de Block LH, Buisman RS, Van Ijzendoorn MH, Elzinga BM. 2018 Pass it on? The neural responses to rejection in the context of a family study on maltreatment. Soc. Cogn. Affect. Neurosci. **13**, 616-627. (10.1093/scan/nsy035) 29897537 PMC6022637

[27] Downey G, Feldman SI. 1996 Implications of rejection sensitivity for intimate relationships. J. Pers. Soc. Psychol. **70**, 1327-1343. (10.1037/0022-3514.70.6.1327) 8667172

[28] Downey G, Freitas AL, Michaelis B, Khouri H. 1998 The self-fulfilling prophecy in close relationships: rejection sensitivity and rejection by romantic partners. J. Pers. Soc. Psychol. **75**, 545-560. (10.1037/0022-3514.75.2.545) 9731324

[29] Jacobs N, Harper B. 2013 The effects of rejection sensitivity on reactive and proactive aggression. Aggress. Behav. **39**, 3-12. (10.1002/ab.21455) 23090847

[30] Twenge JM, Campbell WK, Foster CA. 2003 Parenthood and marital satisfaction: a meta-analytic review. J. Marriage Fam. **65**, 574-583. (10.1111/j.1741-3737.2003.00574.x)

[31] Buckley F, Monks K. 2004 The implications of meta-qualities for HR roles. Hum. Resour. Manag. J. **14**, 41-56. (10.1111/j.1748-8583.2004.tb00132.x)

[32] Hillebrandt H, Sebastian C, Blakemore S. 2011 Experimentally induced social inclusion influences behavior on trust games. Cogn. Neurosci. **2**, 27-33. (10.1080/17588928.2010.515020) 24168421

[33] Syrjämäki AH, Hietanen JK. 2018 Social inclusion, but not exclusion, delays attentional disengagement from direct gaze. Psychol. Res. **84**, 1126-1138. (10.1007/s00426-018-1108-2) 30324264 PMC7239803

[34] van Schie CC, Chiu CD, Rombouts SARB, Heiser WJ, Elzinga BM. 2018 When compliments don't hit but critiques do: an fMRI study into self-esteem and self-knowledge in processing social feedback. Soc. Cogn. Affect. Neurosci. **13**, 404-417. (10.1093/scan/nsy014) 29490088 PMC5928412

[35] Eisenberger NI, Inagaki TK, Muscatell KA, Haltom KEB, Leary MR. 2011 The neural sociometer: brain mechanisms underlying state self-esteem. J. Cogn. Neurosci. **23**, 3448-3455. (10.1162/jocn_a_00027) 21452934

[36] Leary MR, Twenge JM, Quinlivan E. 2006 Interpersonal rejection as a determinant of anger and aggression. Pers. Soc. Psychol. Rev. **10**, 111-132. (10.1207/s15327957pspr1002_2) 16768650

[37] Will G, Rutledge RB, Moutoussis M, Dolan RJ. 2017 Neural and computational processes underlying dynamic changes in self- esteem. Elife **6**, e28098. (10.7554/eLife.28098) 29061228 PMC5655144

[38] vanDellen MR, Campbell WK, Hoyle RH, Bradfield EK. 2011 Compensating, resisting, and breaking: a meta-analytic examination of reactions to self-esteem threat. Pers. Soc. Psychol. Rev. **15**, 51-74. (10.1177/1088868310372950) 20631397

[39] Cook JL, Ouden HEMD, Heyes CM, Cools R. 2014 The social dominance paradox. Curr. Biol. **24**, 2812-2816. (10.1016/j.cub.2014.10.014) 25454588

[40] Benenson JF, Markovits H, Hultgren B, Nguyen T, Bullock G, Wrangham R. 2013 Social exclusion: more important to human females than males. PLoS ONE **8**, e55851. (10.1371/journal.pone.0055851) 23405221 PMC3566112

[41] Stroud LR, Salovey P, Epel ES. 2002 Sex differences in stress responses: social rejection versus achievement stress. Biol. Psychiatry **52**, 318-327. (10.1016/S0006-3223(02)01333-1) 12208639

[42] Cook J, Swart J, Froböse M, Diaconescu A, Geurts D, den Ouden H, Cools R. 2019 Catecholaminergic modulation of meta-learning. Elife **8**, e51439. (10.7554/eLife.51439) 31850844 PMC6974360

[43] Kalma AP, Visser L, Peeters A. 1993 Sociable and aggressive dominance: personality differences in leadership style? Leadership Q. **4**, 45-64. (10.1016/1048-9843(93)90003-C)

[44] Leary MR, Kowalski RM. 1995 The self-presentation model of social phobia. In Social phobia: diagnosis, assessment, and treatment (eds RG Heimberg, MR Liebowitz, DA Hope, FR Schneier), pp. 94-112. New York, NY: The Guilford Press.

[45] Twenge JM, Campbell WK. 2002 Self-esteem and socioeconomic status: a meta-analytic review. Pers. Soc. Psychol. Rev. **6**, 59-71. (10.1207/S15327957PSPR0601_3)

[46] Rosenberg M. 1965 Society and the adolescent self-image. Princeton, NJ: Princeton University Press.

[47] Gray-Little B, Williams VSL, Hancock TD. 1997 An item response theory analysis of the Rosenberg Self-Esteem Scale. Pers. Soc. Psychol. Bull. **23**, 443-451. (10.1177/0146167297235001)

[48] Robins RW, Hendin HM, Trzesniewski KH. 2001 Measuring global self-esteem: construct validation of a single-item measure and the Rosenberg Self-Esteem Scale. Pers. Soc. Psychol. Bull. **27**, 151-161. (10.1177/0146167201272002)

[49] Zimet GD, Dahlem NW, Zimet SG, Farley GK. 1988 The multidimensional scale of perceived social support. J. Pers. Assess. **37**, 1359-1369. (10.1207/s15327752jpa5201_2) 2280326

[50] Barratt W. 2006 The Barratt simplified measure of social status (BSSS): Measure SES. See http://socialclassoncampus.blogspot.com/2012/06/barratt-simplified-measure-of-social.html

[51] Watson D, Friend R. 1969 Measurement of social-evaluative anxiety. J. Consult. Clin. Psychol. **33**, 448-457. (10.1037/h0027806) 5810590

[52] Carleton RN, McCreary DR, Norton PJ, Asmundson GJG. 2006 Brief Fear of Negative Evaluation scale—revised. Depress. Anxiety **23**, 297-303. (10.1002/da.20142) 16688736

[53] R Core Team. 2019 R: a language and environment for statistical computing. Vienna, Austria: R Foundation for Statistical Computing. See https://www.R-project.org/.

[54] Bates D, Maechler M, Bolker B, Walker S. 2015 Fitting linear mixed-effects models using lme4. J. Stat. Softw. **67**, 1-48. (10.18637/jss.v067.i01)

[55] Baumeister RF, Leary MR. 1995 The need to belong: desire for interpersonal attachments as a fundamental human motivation. Psychol. Bull. **117**, 497-529. (10.1037/0033-2909.117.3.497) 7777651

[56] Burke PJ, Stets JE. 1999 Trust and commitment through self-verification. Soc. Psychol. Q. **62**, 347-366. (10.2307/2695833)

[57] Stinson DA, Logel C, Holmes JG, Wood JV, Forest AL, Gaucher D, Fitzsimons GM, Kath J. 2010 The regulatory function of self-esteem: testing the epistemic and acceptance signaling systems. J. Pers. Soc. Psychol. **99**, 993-1013. (10.1037/a0020310) 20822286

[58] Swann WB Jr, Brooks M. 2012 Why threats trigger compensatory reactions: the need for coherence and quest for self-verification. Soc. Cogn. **30**, 758-777. (10.1521/soco.2012.30.6.758)

[59] Marini F, Van den Berg B, Woldorff MG. 2015 Reward-prospect interacts with trial-by-trial preparation for potential distraction. Vis. Cogn. **23**, 313-335. (10.1080/13506285.2015.1023387) 26180506 PMC4500291

[60] Van den Berg B, Krebs RM, Lorist MM, Woldorff MG. 2014 Utilization of reward-prospect enhances preparatory attention and reduces stimulus conflict. Cogn. Affect. Behav. Neurosci. **14**, 561-577. (10.3758/s13415-014-0281-z) 24820263 PMC4412026

[61] Selbing I, Olsson A. 2017 Beliefs about others' abilities alter learning from observation. Sci. Rep. **7**, 16173. (10.1038/s41598-017-16307-3) 29170461 PMC5701038

[62] Dunlap AS, Nielsen ME, Dornhaus A, Papaj DR. 2016 Foraging bumble bees weigh the reliability of personal and social information. Curr. Biol. **26**, 1195-1199. (10.1016/j.cub.2016.03.009) 27133871

[63] Li L, Li KK, Li J. 2019 Private but not social information validity modulates social conformity bias. Hum. Brain Mapp. **40**, 2464-2474. (10.1002/hbm.24536) 30697880 PMC6865577

[64] Charpentier CJ, Iigaya K, O'Doherty JP. 2020 A neuro-computational account of arbitration between choice imitation and goal emulation during human observational learning. Neuron **106**, 687-699. (10.1016/j.neuron.2020.02.028) 32187528 PMC7244377

[65] Pärnamets P, Olsson A. 2020 Integration of social cues and individual experiences during instrumental avoidance learning. PLoS Comput. Biol. **16**, e1008163. (10.1371/journal.pcbi.1008163) 32898146 PMC7500672

[66] Yifrah B, Ramaty A, Morris G, Mendelsohn A. 2021 Individual differences in experienced and observational decision-making illuminate interactions between reinforcement learning and declarative memory. Sci. Rep. **11**, 5899. (10.1038/s41598-021-85322-2) 33723288 PMC7971018

[67] Blackhart GC, Nelson BC, Knowles ML, Baumeister RF. 2009 Rejection elicits emotional reactions but neither causes immediate distress nor lowers self-esteem: a meta-analytic review of 192 studies on social exclusion. Pers. Soc. Psychol. Rev. **13**, 269-309. (10.1177/1088868309346065) 19770347

[68] van Schie C, Cook JL, Elzinga B, Ly V. 2023 The influence of social evaluation on social and non-social learning. OSF. (10.17605/OSF.IO/PZT47) PMC10206450 37234503

[69] van Schie C, Cook JL, Elzinga B, Ly V. 2023 A boost in self-esteem after positive social evaluation predicts social and non-social learning. Figshare. (10.6084/m9.figshare.c.6630961) PMC10206450 37234503

